# *COL6A3* expression in adipose tissue cells is associated with levels of the homeobox transcription factor PRRX1

**DOI:** 10.1038/s41598-020-77406-2

**Published:** 2020-11-19

**Authors:** Simon N. Dankel, Elise Grytten, Jan-Inge Bjune, Hans Jørgen Nielsen, Arne Dietrich, Matthias Blüher, Jørn V. Sagen, Gunnar Mellgren

**Affiliations:** 1 Mohn Nutrition Research Laboratory, Department of Clinical Science, University of Bergen, Bergen, Norway; 2 Hormone Laboratory, Department of Medical Biochemistry and Pharmacology, Haukeland University Hospital, Bergen, Norway; 3 Department of Surgery, Voss Hospital, Bergen Health Trust, Voss, Norway; 4 Department of Surgery, University of Leipzig, Leipzig, Germany; 5 Department of Medicine, University of Leipzig, Leipzig, Germany

**Keywords:** Transcription, Obesity, Pre-diabetes, Type 2 diabetes

## Abstract

Fibrillar collagen COL6α3 in adipose tissue has been associated with obesity, inflammation, insulin resistance and cancer. We here aimed to identify novel transcriptional regulators of *COL6A3* expression. Based on a transcriptome dataset of adipose tissue, we identified strong correlations for 56 genes with *COL6A3* mRNA, including targets of TGF-β/SMAD signaling. Among the identified candidates, the homeobox transcription factor *PRRX1* showed a particularly striking co-expression with *COL6A3*, validated across several different cohorts, including patients with extreme obesity, insulin sensitive and resistant obesity (subcutaneous and omental), after profound fat loss (subcutaneous), and lean controls (subcutaneous). In human and mouse adipose cells, PRRX1 knockdown reduced *COL6A3* mRNA and PRRX1 overexpression transactivated a reporter construct with the endogenous human *COL6A3* promoter. Stable PRRX1 overexpression in 3T3-L1 cells induced *Col6a3* mRNA threefold specifically after adipogenic induction, whereas TGF-β1 treatment upregulated *Col6a3* mRNA also in the preadipocyte state. Interestingly, pro-inflammatory stimulus (i.e., TNF-α treatment) decreased PRRX1-mediated *Col6a3* transactivation and mRNA expression, supporting a role for this mechanism in the regulation of adipose tissue inflammation. In conclusion, we identified the homeobox factor PRRX1 as a novel transcriptional regulator associated with *COL6A3* expression*,* providing new insight into the regulatory mechanisms of altered adipose tissue function in obesity and insulin resistance.

## Introduction

Altered adipose tissue function during chronic energy surplus mediates systemic insulin resistance and disease pathogenesis^1^. The pathogenic potential of adipose tissue partly involves hypertrophied single adipocytes due to insufficient regeneration of new adipocytes, and restrained tissue expandability resulting from increased rigidity of the extracellular matrix (ECM)^1^. Pro-inflammatory macrophages can impair preadipocyte differentiation into mature adipocytes and promote a pro-fibrotic preadipocyte phenotype, in part involving transforming growth factor β (TGF-β) and tumor necrosis factor α (TNF-α) signaling^2,3^. In turn, both impaired adipogenesis and increased collagen deposition may disrupt the normal lipid storage capacity of adipose tissue during energy surplus, and lead to glucotoxicity and lipotoxicity^4,5^.

Collagen constitutes a microfibrillar network composed of several different types of collagen fibrils, including type I and type VI alpha 1–3 (encoded by the genes *COL1* and *COL6A1-3*, respectively). Several studies have implicated *COL6* in altered adipose tissue function in obesity and insulin resistance. For instance, *Col6* knock-out mice were protected from diet-induced metabolic dysregulation despite adipocyte hypertrophy^6^, at least partly dependent on pro-fibrotic and pro-inflammatory effects of a Col6α3-derived signaling peptide called endotrophin acting on adipocytes^7,8^. Adipocyte-derived endotrophin may also promote breast cancer via ECM interactions^9,10^, and circulating Col6α3 may serve as a prognostic marker for cancer^11,12^. In human adipose tissue, consistent with rodent models, up-regulation of *COL6A3* associates positively with obesity-related inflammation, insulin resistance and metabolic dysregulation^13,14^. COL6α3 may partly promote insulin resistance by restricting adipogenesis. We have observed increased *COL6A3* expression in small compared to large adipocytes, and after knockdown of the insulin sensitizing transcription factor peroxisome proliferator activator gamma (PPARγ)^15^. Others also found that knockdown of *COL6A3* in primary human adipocytes suppressed macrophage chemoattractant protein MCP1, supporting pro-inflammatory effects of COL6α3^16^.

Yet, seemingly contradictory to these compelling data, studies have reported decreased adipose *COL6A3* mRNA expression in extreme obesity in the context of high inflammatory gene expression, and increased *COL6A3* mRNA after profound fat loss in the same people^17^, as well as increased *COL6A3* mRNA after a very low-calorie diet^18^. These data indicate a complex regulation of *COL6A3* in adipose tissue dependent on specific metabolic states and transcriptional context. We here sought to improve our understanding of adipose *COL6A3* regulation, which could potentially identify novel therapeutic targets for modulating adipose tissue function. Based on a systematic co-expression analysis for *COL6A3*, together with co-expression patterns in additional cohorts, knockdown experiments and functional transactivation assays, we here discovered paired related homeobox 1 *(PRRX1*) as a novel positive regulator of *COL6A3* expression.

## Results

### Co-expression analysis reveals correlation of COL6A3 and PRRX1 mRNA levels

Co-expression analysis may reveal transcriptional regulators of a target gene of interest. Thus, to probe for potential transcriptional regulators of *COL6A3*, we correlated *COL6A3* mRNA with other mRNA transcripts in our previously published global transcriptome analysis of subcutaneous adipose tissue, which included 16 people with obesity before and after profound fat loss as well as 13 lean people^17^ (Cohort 1, Table 1). This co-expression analysis revealed 56 unique curated genes whose mRNA levels correlated strongly with *COL6A3* mRNA (Pearson’s r > 0.75), thereof 4 inversely correlated genes (Table S2). Among the 52 positively co-expressed genes were several other genes encoding collagens (*COL1A1*, *COL1A2*, *COL3A1* and *COL5A1*).

**Table 1:** Clinical characteristics of subjects included in the study.

	Men/women	Age (years)	BMI (kg/m^2^)	Specific analyses	Figure
**Cohort 1**					
Pre	4/12	39.3 ± 10.9	53.3 ± 4.3	Whole SC/OM	1A-B, S1A
Post	4/12	40.3 ± 10.9	33.1 ± 5.0		
Lean	7/6	44.0 ± 17.5	23.0 ± 2.5		
**Cohort 2**					
ISO	10/20	44.6 ± 1.9	45.1 ± 1.3	Whole SC/OM, euglycemic hyperinsulinemic clamp	1C-D
IRO	10/20	44.9 ± 2.1	45.2 ± 1.3		
**Cohort 3**					
Obese	4/8	43.1 ± 10.4	43.7 ± 5.4	Isolated mature SC/OM adipocytes, SVF	2, S1B
Lean	5/7	43.5 ± 11.6	22.8 ± 2.1		
**hASCs**					
	0/4	44.5 ± 6.6	31.2 ± 5.2	Cultured and differentiated SC SVF	S2A, 3
	1/3	39.0 ± 13.8	24.0 ± 1.4	siRNA knockdown experiments	

BMI, body-mass index (kg/m^2^); hASCs, human adipose stromal cells; IRO, insulin resistant obese; ISO, insulin sensitive obese; OM, omental adipose tissue; Pre, before bariatric surgery; Post, one year after bariatric surgery/after profound fat loss; SC, subcutaneous adipose tissue; SVF, stromal vascular fraction.

**Figure 1:**
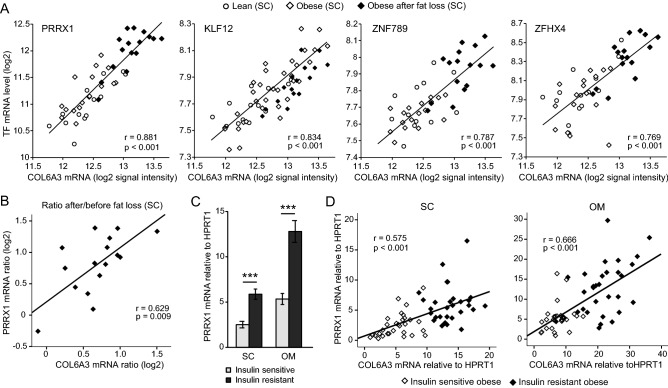
*PRRX1* and *COL6A3* mRNA correlate in human adipose tissue. (**A**) Adipose tissue biopsies were obtained from extremely obese subjects (subcutaneous n = 16, omental n = 12). Subcutaneous adipose tissue was also obtained from the same subjects after profound fat loss (one year after bariatric surgery, n = 16), and from healthy non-obese subjects (n = 14). Gene expression was measured by Illumina microarrays, log2-transformed expression values are shown. Pearson correlations (r) of *COL6A3* mRNA and the mRNA expression of identified potential transcriptional regulators of *COL6A3* are shown. (**B**) Correlation between the change of *PRRX1* mRNA and change of *COL6A3* mRNA after fat loss in 16 pairs of subcutaneous adipose tissue samples collected before and one year after bariatric surgery. (**C**) *PRRX1* mRNA expression in subcutaneous and omental adipose tissue in 30 morbidly obese insulin sensitive and 30 BMI-matched morbidly obese insulin resistant people, measured by qPCR and calculated relative to *HPRT1* mRNA. Insulin sensitivity was measured by euglycemic hyperinsulinemic clamp. (**D**) Correlation of relative *PRRX1* and COL6A3 mRNA across the BMI-matched morbidly obese insulin sensitive and resistant groups, for subcutaneous and omental adipose tissue, respectively. SC, subcutaneous; OM, omental. ***p value < 0.001.

To gain insight into the transcriptional regulation of the set of 56 *COL6A3*-co-expressed genes as a whole, we interrogated a database of known transcription factor (TF)-target gene interactions (TFactS)^19^. The *COL6A3*-associated gene set showed a particularly strong enrichment for targets of SMAD3 (4 genes), SMAD7 (4 genes), TFAP2A (4 genes) and SPI1 (3 genes), including *COL6A3* and other collagen genes identified as targets of SMAD3 and SMAD7 (Tables S3-4). *COL1A1*, *COL1A2*, *COL3A1* and *COL5A1* were also predicted targets of several other TFs (Table S4). SMAD TFs mediate signaling by the TGF-β superfamily of ligands, including bone morphogenic proteins (BMPs), growth and differentiation factors (GDFs) and TGF-βs, which play a critical role in development and homeostasis from embryogenesis through adulthood^20^. TGF-β signaling has been shown to prevent downregulation of collagen genes in fibroblasts and to impair adipogenesis^21,22^. However, none of the genes encoding these TFs showed co-expression with *COL6A3* in our dataset. To prioritize potentially novel TFs that may contribute importantly to the regulation of *COL6A3* expression, we used a curated list of transcription factors^23^ and identified four TF-encoding genes among the 56 top *COL6A3* co-expressed genes (*PRRX1*, *KLF12*, *ZNF789* and *ZFHX4*). Among these, *PRRX1* showed the strongest correlation (Pearson’s r = 0.881) and highest expression level in the adipose tissue (Fig. 1A). The strong positive correlation for *PRRX1* and *COL6A3* was evident within each subgroup (subcutaneous fat in the lean, obese and obese after fat loss, and omental fat in the obese) (Figure S1A), and also for the change in *PRRX1* mRNA in relation to the change in *COL6A3* mRNA after profound fat loss (Fig. 1B). These data revealed a striking correlation between *PRRX1* and *COL6A3* mRNA levels in adipose tissue.

By qPCR we further sought to validate the co-expression of *PRRX1* and *COL6A3* in adipose samples from additional cohorts, including different adipose depots and isolated human adipocytes and stromal vascular fraction (SVF). We previously reported increased *COL6A3* mRNA in insulin resistant compared to insulin sensitive obese patients^15^. These patients were otherwise healthy and matched for age, sex, BMI and total body fat^24^. Similar to *COL6A3* in these patients (Cohort 2, Table 1), *PRRX1* showed 2–threefold higher expression in both subcutaneous and omental adipose tissue in the insulin resistant compared to the insulin sensitive patients (Fig. 1C,D). Consistent with the initial cohort, each of the depots showed a strong positive correlation between *PRRX1* and *COL6A3* mRNA (Fig. 1D).

**Figure 2:**
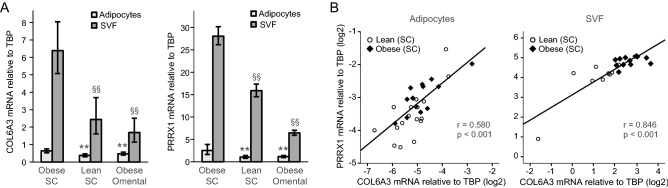
Expression of *COL6A3* and *PRRX1* in mature adipocytes and stromal vascular fraction (SVF). Adipocytes and SVF from human subcutaneous and omental whole tissue biopsies were separated by collagenase treatment, and gene expression was measured by qPCR using *TBP* as a reference gene. (**A**) The expression of *PRRX1* was measured in subcutaneous adipocytes and SVF from morbidly obese people (n = 13, SVF n = 12), subcutaneous adipocytes in non-obese subjects (adipocyte n = 17, SVF n = 10), and omental adipocytes in severely obese subjects (adipocyte n = 13, SVF n = 12). Data are presented as mean ± SEM. (**B**) *PRRX1* and *COL6A3* mRNA were correlated in mature subcutaneous adipocytes and SVF (log2-transformed data, Pearson correlation). SC, subcutaneous; SVF, stromal vascular fraction. **p value < 0.01 (relative to obese SC for adipocytes); §§p value < 0.01 (relative to obese SC for SVF).

Comparing adipocytes and SVF in another cohort (Cohort 3, Table 1), the two genes showed a highly similar expression pattern across lean and obese subcutaneous and obese omental samples, with highest expression in subcutaneous SVF in obesity (Fig. 2A). This corresponded to strong positive correlations in both adipocytes and SVF (Fig. 2B). Also the omental samples (available from the 12 patients with obesity) showed positive correlations of *PRRX1* and *COL6A3* mRNA (SVF: p = 8.66E-5, *r* = 0.894; adipocytes: p = 0.263, *r* = 0.335, data now shown). The microarray analysis showed considerably lower expression levels for *KLF12*, *ZNF789* and *ZFHX4*, with similar expression in adipocytes and SVF, in contrast to *COL6A3* and *PRRX1* (Figure S1B). Together, these data show a consistent co-expression of *PRRX1* and *COL6A3* in adipose tissue across different phenotypic contexts and nutritional states, suggesting that PRRX1 might contribute to the transcriptional regulation of *COL6A3*.

We and others previously identified PRRX1 as a suppressor of adipogenesis^25,26^. We here assessed the expression profiles of *PRRX1* and *COL6A3* during adipogenic differentiation. In primary human adipose cultures, neither gene showed a notable change in expression throughout differentiation (Figure S2A). Increased expression of *PPARG* variant 2 and morphological analysis confirmed that the cells differentiated into adipocytes (Figure S2A). In 3T3-L1 mouse adipocytes, *Col6a3* mRNA showed fluctuating expression although there was notable variation across experiments (Figure S2B). On the other hand, *Prrx1* mRNA consistently showed a marked decrease throughout differentiation. Both genes showed relatively stable expression in undifferentiated cells grown in parallel with differentiated cells (Figure S2B). Increased *Pparg* variant 2 expression confirmed differentiation in the 3T3-L1 cells (Figure S2B).

### PRRX1 transactivates* COL6A3*

**Figure 3:**
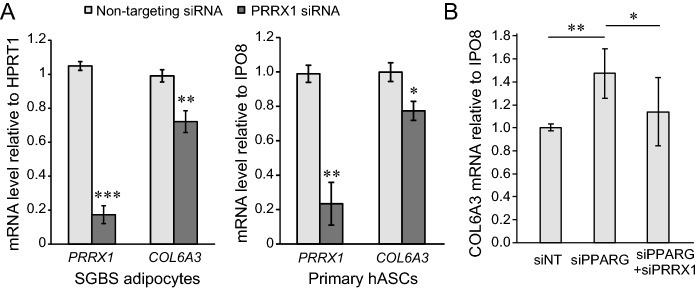
PRRX1 knockdown reduces *COL6A3* mRNA expression in human adipose cells. The day after seeding (70–80% confluence) in 12-well plates, cells were transfected with siRNA (25 nM for *PRRX1* and 10 nM for *PPARG*), induced to differentiate, and left for 72 h. *COL6A3* mRNA expression was measured by qPCR normalized to *HPRT1* mRNA (SGBS) or *IPO8* mRNA (hASCs). (**A**) Data for human SGBS preadipocytes presented as mean ± SD for an experiment performed in triplicate. (**B**) hASCs were isolated from adipose tissue liposuction aspirate of four people. Data are presented as mean ± SD for four independent experiments performed in triplicate. hASCs, primary human adipose stromal cells, siNT, non-targeting siRNA (control). *p value < 0.05; **p value < 0.01; ***p value < 0.001.

To assess whether PRRX1 may be a novel transcriptional regulator of adipose *COL6A3* expression, and not merely co-expressed, we next silenced *PRRX1* in human adipose cells and measured endogenous *COL6A3* mRNA expression. When differentiating SGBS or primary human preadipocytes for 3–4 days, we found that knock-down of *PRRX1* mRNA led to 20–30% reduction in *COL6A3* mRNA (Fig. 3A).

We previously observed that knockdown of *PPARG* increased *COL6A3* mRNA in differentiating primary human adipocytes, indicating that PPARγ is a negative regulator of *COL6A3* mRNA^15^. Furthermore, in a separate study we demonstrated that PRRX1 inhibits *PPARG* mRNA expression in the early phase of adipogenic differentiation^25^. We here tested if *PRRX1* knockdown would counteract the upregulation of *COL6A3* mRNA after *PPARG* knockdown in early adipogenesis. As hypothesized, *PRRX1* silencing significantly blunted the upregulation of *COL6A3* mRNA caused by *PPARG* knockdown (Fig. 3B). Taken together, these data indicate that increased expression of *COL6A3* in these cells at least partly depends on the simultaneous presence of PRRX1 and reduction in PPARγ.

**Figure 4:**
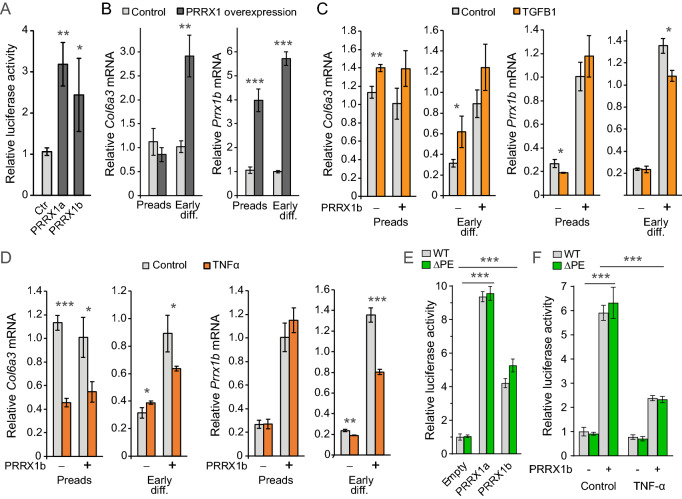
PRRX1 transactivates the human *COL6A3* promoter. (**A**) 3T3-L1 cells were transfected with expression plasmid encoding PRRX1a or PRRX1b together with a luciferase construct containing part of the endogenous human *COL6A3* promoter with a predicted PRRX1 binding element. Data from 5 independent experiments performed in triplicate were combined and normalized. (**B–D**) A 3T3-L1 cell line stably overexpressing Prrx1b and a corresponding control 3T3-L1 cell line were grown to 100% confluence. Cells that were induced to differentiate into adipocytes were grown for an additional 2 days before treatment with adipogenic compounds for 2 days. The cells were treated with vehicle, TGF-β1 (1 ng/ml) or TNF-α (10 ng/ml) for the last 24 h before lysis. *Col6a3* and *Prrx1b* mRNA were measured by qPCR and calculated relative to *Rplp0* mRNA. Experiments were performed in triplicates. (**E**) COS-1 cells were transfected with a luciferase reporter construct under control of either the wild-type (WT) *COL6A3* promoter or the same promoter with a deletion in the predicted PRRX1 binding element (ΔPE) together with an overexpression plasmid encoding either PRRX1a or PRRX1b. An empty overexpression plasmid was used as negative control. (**F**) COS-1 cells transfected as previously with WT and ΔPE *COL6A3* luciferase reporter in combination with PRRX1b, in addition to treatment with vehicle or TNF-α (100 ng/ml). Representative of two independent experiments. Data are presented as mean ± SD. Early diff., early differentiation, i.e., cells induced to differentiate into adipocytes for two days; Preads, preadipocytes; WT, wild-type; ΔPE, deletion of predicted PRRX1 binding element. *p value < 0.05; **p value < 0.01; ***p value < 0.001.

We consequently tested the transcriptional regulation of *COL6A3* by PRRX1 overexpression. We first performed transient transfection of a vector expressing PRRX1 and a luciferase reporter construct containing part of the human *COL6A3* promoter (COL6A3-PROM-luc) and a predicted PRRX1 binding motif (Figure S3). When transfecting this reporter into 3T3-L1 cells together with either of the PRRX1 isoforms (PRRX1a and PRRX1b), we found a 2.5 to threefold increase in luciferase activity (p < 0.001) (Fig. 4A). To examine if PRRX1 also induces endogenous *COL6A3* mRNA expression, and whether this varies with different cellular contexts, we analyzed mRNA in 3T3-L1 preadipocytes with stable PRRX1b overexpression, with and without adipogenic induction. Interestingly, while PRRX1 overexpression did not affect *Col6a3* mRNA in preadipocytes before differentiation relative to control cells, upon adipogenic induction the PRRX1-overexpressing cells showed a threefold increase in endogenous *Col6A3* mRNA expression (Fig. 4B).

As our transcriptome screen linked *COL6A3* co-expressed genes to TGF-β/SMAD signaling (e.g., SMAD3 and SMAD7), in line with published literature showing enhanced collagen gene expression upon TGF-β stimulation in various cell types^27^, we next tested if TGF-β might induce *Col6a3* mRNA at least in part via Prrx1. As expected, TGF-β treatment increased *Col6a3* mRNA in preadipocytes, and also in early adipogenic differentiation (Fig. 4C). PRRX1 overexpression added to the positive effect of TGF-β, although only in cells that were induced to differentiate (Fig. 4C). However, the TGF-β-stimulated *Col6a3* expression was at least partly independent of changes in *Prrx1* expression, since some of the cells treated with TGF-β showed reduced *Prrx1* mRNA (Fig. 4C). These data suggest that PRRX1 did not act principally through TGF-β-dependent SMAD3 and SMAD7 transcriptional activity to induce *COL6A3* mRNA.

Inflammation represents another pathway in which PRRX1 might mediate increased *COL6A3* expression, as *COL6A3* has been implicated in adipose tissue inflammation^13,16^, including a role in anti-inflammatory M2-type macrophages^14^. However, it is unknown whether inflammatory factors such as TNF-α modulate *COL6A3* expression in adipose cells, and whether PRRX1 might be involved in this. Based on our previous finding that both *PRRX1* and *COL6A3* showed increased adipose expression after bariatric surgery in people with extreme obesity concomitant with markedly reduced inflammation^17^, we hypothesized that TNF-α reduces *COL6A3* mRNA, at least in part via reduced PRRX1. When treating 3T3-L1 cells with TNF-α we found 25 – 60% reduction in *Col6a3 mRNA* regardless of whether Prrx1 was overexpressed or not (Fig. 4D). In line with the stimulatory effect of Prrx1 overexpression on *Col6a3* mRNA specifically in cells that were induced to differentiate, as opposed to preadipocytes (Fig. 4B,D), the decreased *Col6a3* mRNA overall corresponded to decreased *Prrx1* mRNA in cells induced to differentiate and not in preadipocytes (Fig. 4D). Of note, in the absence of Prrx1 overexpression, TNF-α increased *Col6a3* mRNA slightly in control cells that were induced to differentiate while decreasing Prrx1 mRNA in these cells, suggesting also Prrx1-independent regulation (Fig. 4D). Interestingly, TNF-α also markedly blunted the *Prrx1* mRNA level in the cells that were transfected to overexpress Prrx1. While the reason for this effect is unclear, the decreased *Prrx1* mRNA nonetheless corresponded to decreased *Col6a3* mRNA (Fig. 4D).

Although our data clearly show that PRRX1 induces the transcription of *COL6A3* mRNA in differentiating adipose cells, and that TNF-α stimulation can blunt this effect of PRRX1 at least in part by reducing *PRRX1* expression, it is not clear if PRRX1 regulates *COL6A3* expression by direct binding to the *COL6A3* promoter or indirectly via regulation of other endogenous factors. To examine this, we mutated the core of the PRRX1 binding motif in the *COL6A3*-PROM-luc construct, and tested the effect of PRRX1 overexpression with and without TNF-α in COS-1 cells, in which a PRRX1-mediated transactivation may be less dependent on the endogenous context of 3T3-L1 cells. Interestingly, while both Prrx1a and Prrx1b strongly increased luciferase activity as expected, the disrupted binding motif did not alter the PRRX1-mediated transactivation potential (Fig. 4E). However, consistent with the inhibitory effect of TNF-α on *Col6a3* and *Prrx1* mRNA in differentiating 3T3-L1 cells, TNF-α potently inhibited the stimulatory effect of PRRX1 overexpression on *COL6A3* promoter activity (Fig. 4F).

## Discussion

The regulation of adipose tissue function has gained considerable interest in recent years, with the realization that adipose tissue is an endocrine organ contributing to development of chronic diseases such as type 2 diabetes and cancer^1^. COL6α3 is an important extracellular matrix (ECM) component in adipose tissue, showing altered expression in different metabolic contexts and cell types, and with a potential causal role in obesity-related metabolic diseases^13,14,16^. Adipose *COL6A3* expression appears highly dynamic and context-dependent, as different studies have reported elevated levels upon body weight gain^13^ as well as after bariatric surgery^17^ and diet-induced weight loss^18^. Importantly, previous studies have shown cell-type dependent expression of *COL6A3*, with obesity-dependent expression in adipocytes^15^ but higher expression in the stromal vascular fraction (SVF) of adipose tissue compared to isolated adipocytes^18^, expression across different adipose tissue resident monocytes/macrophages (e.g., in a nondestructive, ECM-conserving^29^ and potentially pro-fibrotic subtype^14^), and expression in different tumor cells^30,31^. Heterogeneity in cellular phenotypes of adipose tissue in different physiological contexts may therefore explain the variable results observed for *COL6A3* expression in different cohorts and interventions of people with obesity. Transcriptional regulation of *COL6A3* expression by PRRX1 helps to elucidate its dynamic expression, and may represent a new treatment target to modulate *COL6A3* expression in COL6α3-dependent pathological conditions.

Our findings link PRRX1 to adipose tissue fibrosis, which associates with insulin resistance in humans^32^, and in which COL6α3/endotrophin may play an important causal role^7,10^. PRRX1 is a developmental homeobox transcription factor, and has an established role in embryonic and postnatal skeletogenesis^33,34^. One of few established target genes of PRRX1 is tenascin C (TNC)^35,36^, a pro-fibrotic factor involved in a Twist1-Prrx1-TNC positive feedback loop in fibroblast activation^37^. Recent studies have revealed pro-fibrotic effects of PRRX1 in hepatic stellate cells^38^ and lung fibroblasts^39^. In hepatic stellate cells, PRRX1 was shown to transactivate the *COL1A1* promoter^40^. PRRX1-mediated regulation of *COL6A3* may however be of particular importance, since *COL6A3* overexpression stimulates TGF-β signaling and increases expression of other collagen genes^7^.

Here we found that TGF-β increased *Col6a3* mRNA expression in adipose cells, as could be expected. It is possible that PRRX1 at least partly increased *COL6A3* expression via TGF-β, since a suppressive effect of PRRX1 on adipogenesis was previously found to involve TGF-β signaling^26^. Together with the positive effect also of COL6α3 on TGF-β signaling^7^, PRRX1 might be involved in a positive feedback loop between TGF-β and COL6A3, at least in certain cellular contexts. However, our data overall suggest that PRRX1 may induce *COL6A3* mRNA independently of TGF-β/Smad signaling, since we found that TGF-β up-regulated *Col6a3* mRNA while decreasing or not changing *Prrx1* mRNA levels in 3T3-L1 cells.

The stimulatory effect of PRRX1 overexpression on *Col6a3* mRNA specifically in preadipocytes that were induced to differentiate, and not in cells still in the preadipocyte state, suggests that the relationship between PRRX1 and *COL6A3* involves factors in adipocyte development. A PRRX1-*COL6A3* axis may be important in adipogenesis and adipose tissue function related to PPARγ signaling. We previously found that PRRX1 directly inhibits *PPARG2* expression and represses adipogenesis via a causal genetic variant that predisposes to type 2 diabetes^25^. Consistent with the inhibitory effect of PRRX1 on adipogenesis via TGF-β signaling^26^, TGF-β-induced dedifferentiation of human adipocytes decreases *PPARG* expression while increasing *TGFB1* and *COL6A3* expression^41,42^. Here we additionally showed, during early differentiation of primary human adipose stromal cells, that knockdown of PRRX1 dampens the increase in *COL6A3* expression seen after PPARG knockdown. Thus, PRRX1 may, at least in part, enhance *COL6A3* expression indirectly via suppression of *PPARG2* expression. Furthermore, knockdown of *COL6A3* in immortalized primary human preadipocytes has been found to increase expression of adipogenic genes^16^, supporting that PRRX1 inhibits adipogenesis via regulation of both *PPARG2* and *COL6A3*. Whether additional factors mediate the effect of altered PRRX1 levels on *COL6A3* expression during adipogenesis, and the reason why PRRX1 overexpression had no effect on *Col6a3* mRNA in preadipocytes in our experiments, requires further investigation.

We further gained new insight into the regulation of *COL6A3* by pro-inflammatory stimuli. The down-regulation of *Col6a3* mRNA upon TNF-α treatment in 3T3-L1 cells is consistent with *COL6A3* down-regulation in the context of inflamed adipose tissue from people with obesity, and up-regulation after bariatric surgery and diet-induced weight loss^17,18^. Moreover, it is conceivable that reports of increased adipose *COL6A3* expression in obesity^13,15,16^ might not reflect effects of pro-inflammatory stimuli, but the activity of M2-type macrophages with pro-fibrotic but anti-inflammatory properties^14^. Taken together, our data support that *COL6A3* expression is positively regulated by signals involved in tissue remodeling and potentially fibrosis (e.g., TGFB1), while being negatively regulated by at least some pro-inflammatory stimuli.

Because several tissues express both *COL6A3* and *PRRX1*, our discovery of PRRX1 as an upstream transcriptional regulator of *COL6A3* may be relevant not only for adipose tissue. *COL6A3* has been implicated in pathogenesis of the nervous system, muscular dystrophy and isolated dystonia (a disorder of involuntary muscle twitching)^43,44^, and in different forms of cancer including of the pancreas^30^, bladder^31^, colon^11^, ovary^45^ and breast^10^ (the latter via adipose tissue). Fibrosis plays an important although complex role also in cancer, in part by characterizing solid tumors^46^. The adipose-COL6α3-cancer link involves the epithelial-to-mesenchymal transition (EMT)^10^, modulated by the TGF-β/Smad pathway^31^ and which can be induced by adipocytes^47,48^. EMT allows formation of mesenchymal tissues distant from the originating epithelial cells in cancer as well as in embryogenesis. EMT further plays a role in reversible transdifferentiation of epithelial cells into adipocytes in mammary tissue^49^ and PPARγ2-mediated conversion of invasive breast cancer cells into fat-storing cells^50^. PRRX1 is an established regulator of EMT in cancer cells^31,51^, supporting that PRRX1 may regulate *COL6A3* during EMT. Interestingly, the PRRX1b isoform may promote EMT and cancer cell invasion, while the PRRX1a isoform may promote the reverse (mesenchymal-to-epithelial transition, MET) and metastatic outgrowth^52^. Taken together, our data suggest that PRRX1 may be a target in adipocytes and potentially other cell types to modulate *COL6A3* expression in the context of pathological conditions including cancer.

Our study has important limitations. Although the *COL6A3* reporter contained a predicted consensus PRRX1 binding site, and the PRRX1 knockdown and overexpression clearly affected endogenous *COL6A3* expression, our data do not support that PRRX1 regulates *COL6A3* mRNA by direct binding to the *COL6A3* promoter. Rather, the regulation may be indirect by hitherto unknown endogenous factors that are regulated by PRRX1 on the transcriptional and/or posttranscriptional level. Despite our efforts we were unable to provide a mechanistic explanation for the striking and highly consistent correlations between *PRRX1* and *COL6A3* mRNA that we observed in several human cohorts. Moreover, we cannot readily explain the seemingly contradictory expression patterns in different cohorts reported here or in the literature, where *PRRX1* and *COL6A3* were downregulated in obesity as well as upregulated after fat loss in some cohorts, while being upregulated in insulin resistant relative to insulin sensitive subjects in others. Moreover, we did not assess protein expression to demonstrate that altered mRNA corresponds to altered protein levels, and to support that PRRX1 modulates COL6α3 function. Finally, although our strategy to identify transcriptional regulators of *COL6A3* coupled with overexpression assays revealed PRRX1 as a potent regulator of *COL6A3 *in vitro, and these factors showed strong joint correlations with obesity and insulin resistance, the physiological relevance of this relationship remains to be demonstrated in vivo.

## Conclusion

In conclusion, through correlation analyses based on different human cohorts, consistent co-expression patterns and PRRX1 knockdown, overexpression and transactivation experiments, we have identified PRRX1 as a novel transcriptional regulator of *COL6A3* mRNA expression in adipose cells. The PRRX1-*COL6A3* axis, and modulators thereof yet to be discovered, may represent promising treatment targets for mitigating obesity-related pathogenesis, including insulin resistance and cancer. Interestingly, the present study identified TNF-α as a novel inhibitor of PRRX1-mediated transactivation of the *COL6A3* promoter. Future studies should further dissect the mechanisms by which this and additional upstream pathways regulate the PRRX1-COL6A3 relationship in adipose and other tissues.

## Methods

### Ethical statement

The study was approved by the Regional Committee for Medical Research Ethics in Western Norway (REK Vest, approval numbers 2010/512 and 2010/3405), and the ethics committee of the University of Leipzig (approval number 159–12-21,052,012 and 017–12-23,012,012). Each subject gave written informed consent. We carried out all methods in accordance with relevant guidelines and regulations.

### Subjects and biopsy for gene expression analysis

Subcutaneous (SC) and omental (OM) adipose tissue biopsies were obtained by surgical excision from Caucasian patients with severe obesity undergoing bariatric surgery in Western Norway (Førde Hospital and Voss Hospital) or in Leipzig, Germany, as previously described^17,24,53^ (Cohorts 1–3, Table 1). Subcutaneous biopsies were also obtained from a subset of patients one year after the bariatric surgery and from non-obese healthy people^17^ (Cohort 1). In Cohort 2^24^, the subjects were dichotomized into groups of insulin sensitive obese (ISO) and insulin resistant obese (IRO) based on glucose infusion rate < 60 and > 70 μmol/kg/min, respectively. These patients were otherwise healthy and matched for age, sex, BMI and total body fat. In Cohort 3^53^, adipocytes and the stromal vascular fraction (SVF) were isolated from SC adipose tissue of patients undergoing hernia repair (lean/overweight) or bariatric surgery (obese). The tissue was frozen immediately in liquid nitrogen and stored at –80ºC. For primary human adipose culture, liposuction aspirate from abdominal SC adipose tissue was collected at Klinikk Bergen, Norway, and processed the same day.

### Adipose tissue homogenization and fractionation

Frozen whole tissue (200-300 mg) was homogenized in a 2 ml safe-lock eppendorf tube with 1 ml Qiazol lysing buffer (Qiagen) and a 5 mm metal bead (Millipore), using a TissueLyser II (Qiagen) with three repeated shakings at 25 Hz for 2 min each. To isolate adipocytes and SVF, 700-800 mg of adipose tissue was treated with collagenase and thermolysin (Liberase Blendzyme 3, Roche) for 30 min at 37ºC, followed by washing with PBS and careful centrifugation, as previously described^53^. Cells were lysed in Qiazol within one hour ± 5 min and frozen in liquid nitrogen.

### Cell lines

All cells were kept in a humidified CO_2_ incubator at 37˚C with 5% CO_2_ saturation. 3T3-L1 mouse preadipocytes were cultured in high-glucose (4.5 g/L/5 mM) DMEM with 1% penicillin and streptomycin (PEST), and 10% calf serum (CS) during proliferation. The cells were induced to differentiate into adipocytes two days post-confluence (“day 0”), by a two-day treatment with dexamethasone (0.5 mM), insulin (175 nM), phosphodiesterase inhibitor 3-isobutyl-1-methylxanthine (IBMX) (0.5 M) and fetal bovine serum (FBS) (10%). Thereafter, the medium contained 10% FBS and 175 nM insulin, as well as 1 μM rosiglitazone (PPARγ agonist). SGBS preadipocytes were proliferated in DMEM-F12 medium containing 10% fetal calf serum (FCS) and 1% PEST. Differentiation was induced at 90–100% confluence by washing the cells with pre-warmed PBS repeatedly, and by culturing in serum-free medium (2/3 DMEM-F12 and 1/3 MCDB-131 supplemented with 1% PEST, 10 µg/ml transferrin, 66 nM insulin, 100 nM cortisol, 1 nM T3, 0.5 nM IBMX, 25 nM dexamethasone and 2 µM rosiglitazone). After 3 days of incubation, the induction medium was replaced with differentiation medium (2/3 DMEM-F12 and 1/3 MCDB-131 supplemented with 1% PEST, 10 µg/ml transferrin, 66 nM insulin, 100 nM cortisol and 1 nM T3). COS-1 monkey kidney cells were grown in high-glucose (4.5 g/L/5 mM) DMEM, supplemented with 1% penicillin and streptomycin (PEST) and 10% fetal bovine serum (FBS).

### Retroviral infection and stable overexpression

Human PRRX1b (NM_022716.4) was cloned into a retroviral pZOME vector carrying the puromycin resistance gene. 5 µg of constructs were transfected with plasmids carrying vsvg and gagpol into 293et cells using Transfectin transfection reagent (Bio-Rad). 48 h after transfection, the virus particles were harvest by filtering the medium through 0.45 µm syringe filter and were stored at -80ºC. The day before infection, mouse 3T3-L1 cells were plated into 6-well plates. The next day the medium was removed and virus-containing medium was added in the presence of polybrene (4 µg/ml). Two days after infection the cells successfully transduced with virus were selected with puromycin (2 µg/ml). A comparable control cell line expressing green fluorescence protein (GFP) was also generated.

### Primary human adipose culture

Human adipose stromal cells were isolated from liposuction aspirate as described previously^54^. Tissue was digested in 50 ml NUNC tubes for about 2 h at 37ºC, with a 1:1 ratio of tissue and KRP buffer (0.1% BSA and ~ 55 Wunch/liter collagenase with thermolysin (Liberase Blendzyme Thermolysin Medium 10X, Roche)). The tissue was then filtered through a 210 μm nylon mesh into a 125 ml cup, and cells were collected from underneath the floating adipocytes followed by centrifugation at 200 g for 10 min. Adipocytes were washed two more times with 15 ml PBS to release cells, each time followed by collection and centrifugation of the cells. Red blood cells were lysed by treating the cells for 10 min with a buffer containing 155 mM ammonium chloride, 5.7 mM dipotassium phosphate and 0.1 mM EDTA, followed by centrifugation at 200 g for 10 min. Finally, the cells were filtered through a 70 μm nylon mesh cell strainer (BD Falcon), counted using a Bürker chamber, and cultured in 6-well plates (~ 500,000 cells/well) with DMEM GlutaMax (GIBCO) containing 10% FCS and 1% PEST. Cells were differentiated by supplementing the medium with cortisol (100 nM/L), insulin (66 nM/L), transferrin (10 μg/ml), biotin (33 μM), pantothenate (17 μM/L) and T3 (1 nM/L) the day after seeding (day 0), changing medium every 2–3 days. For the first six days, rosiglitazone (10 μM) was also added.

### Gene knock-down by siRNA

Gene silencing by small interfering RNA (siRNA) was performed as previously described^15^. On day 0, the primary human adipose cells were treated with differentiation medium and 25 nM ON-TARGETplus human siRNA SMARTpool (Dharmacon) using HiPerFect (Qiagen) (non-targeting (NT) control and siRNA against PRRX1), according to the manufacturer’s protocol. Cells were collected in buffer RLT (Qiagen) after 72 h and stored at -80ºC until RNA extraction and PCR.

### RNA extraction, cDNA synthesis and qPCR

The procedures were described previously^17^. The RNeasy Lipid Tissue Midi Kit (whole tissue) or Mini Kit (Qiagen) was used to extract total RNA, and NanoDrop ND-1000 spectrophotometer (NanoDrop Technologies) was used to measure yield and quality. For whole tissue, cDNA was prepared from 1 μg total RNA by the Transcriptor First Strand cDNA Synthesis Kit (Roche), followed by 1:10 dilution with PCR-grade water. For the cell fractions and cell culture, the SuperScript VILO cDNA Synthesis Kit (Invitrogen) was used according to the manufacturer’s protocol, with an input of 100 ng (cell fractions) and 500 ng (cell culture) total RNA per sample, followed by 1:20 dilution of the cDNA with PCR-grade water. Standard curves (1:5 or 1:10 dilutions) were made by synthesizing cDNA from 2.5 μg total RNA extracted from whole tissue or cell culture, using the SuperScript VILO kit. The LightCycler480 Probes Master kit and the LightCycler480 rapid thermal cycler system (Roche) were used to perform qPCR. Target and reference genes were amplified by specific primers and Universal ProbeLibrary (UPL) probes (Roche) shown in Table S1. Amplification efficiency based on the standard curves was used to calculate mRNA concentrations. *TBP* and *IPO8*^55^ were chosen as reference genes based on their stable expression in adipose tissue and primary culture, respectively, and because they showed similar expression levels as the target genes. In Cohort 2 the reference gene was *HPRT1*.

### Microarray gene expression analysis

The samples were prepared and microarray analyses performed as described previously^17^. Briefly, 300 ng of total RNA from each sample was reversely transcribed, amplified and Biotin-16-UTP–labeled. NanoDrop spectrophotometer and Agilent 2100 Bioanalyzer were used to measure amount (15–52 mg) and quality of the labeled cRNA. 750 ng of biotin-labeled cRNA was hybridized to the HumanRef-8v.3 (whole tissue) or HumanHT-12v.3 Illumina Sentrix BeadChip according to manufacturer’s instructions. The HumanRef-8v.3 BeadChip targets approximately 24,500 annotated RefSeq transcripts and covers 18,631 unique curated genes. The HumanHT-12v.3 BeadChip targets approximately 48,800 annotated RefSeq transcripts and covers 27,455 unique curated genes. The microarray data are MIAME compliant and are available in ArrayExpress (accession E-TABM-862). The HumanHT-12v.3 BeadChip was also used for the PRRX1 knockdown experiment in 8 primary human adipose cell cultures as described previously^25^.

### Transient transfection and luciferase assay

The *COL6A3* promoter reporter construct (pLightSwitch_Prom) was purchased from SwitchGear Genomics. The PRRX1a and PRRX1b plasmids were gifted by Michael J. Kern. Lipofectamine 2000 (Invitrogen) was used for transfecting 3T3-L1 cells with plasmid DNA, according to the manufacturer’s protocol. Cells were seeded the day before transfection in 24-well plates. Confluence at time of transfection was 70–80%. Prior to transfection, medium was removed and cells were washed with pre-heated (37ºC) PBS followed by addition of 400 µl serum-free medium. DNA was diluted in 100 µl Opti-MEM I Reduced Serum Medium (Invitrogen) and mixed by pipetting. Lipofectamine 2000 was mixed and combined with 100 µl Opti-MEM, before combining µl Lipofectamine and µg DNA at a 1:1 ratio.

48 h after transfection, growth medium was removed and cells were rinsed with 4ºC PBS. The plate was placed on ice and 80 µl lysis buffer was dispensed in each well. To ensure complete lysis the plate was placed on a shaker at 200-250 rpm for 30–40 min at 4ºC. The lysates were then transferred into a 96-well plate and immediately placed on ice, before centrifugation for 5 min at 4600 rpm. 35 µl of the supernatant was transferred to a 96-well plate containing luciferin and ATP substrate, before immediate measurement in a luminometer.

### Mutagenesis of the COL6A3-prom-luc reporter

The following mutagenesis primers targeting the predicted PRRX1 binding site was designed using Agilent’s QuikChange Primer Design software (https://www.agilent.com/store/primerDesignProgram.jsp): Forward primer, 5′-TTGGTTAACAGAAAACCAAGGCGATTT***TT***TGCTGGTTTTTCTATTT-3′; reverse primer, 5′-AAATAGAAAAACCAGCA***AA***AAATCGCCTTGGTTTTCTGTTAACCAA-3′. Bold-Italic letters indicate position of mutation site, flanking the two deleted base pairs (shown in lowercase) from the PRRX1 core binding motif (T***T***aa***T***TG). Mutagenesis was performed using the QuikChange II Site-Directed Mutagenesis Kit (Agilent) according to manufacturer’s instructions. Briefly, WT plasmid was amplified by PCR using the mutagenesis primers and a high-fidelity DNA polymerase, followed by DpnI digestion of methylated template DNA. The mutated plasmid was transformed in TOP10 competent *E.coli* cells (Invitrogen) and isolated using the HiSpeed Plasmid Maxi kit (Qiagen) before verification by sequencing.

### TFactS analysis

TFactS analysis (https://www.tfacts.org/TFactS-new/TFactS-v2/) was run with the following parameters: Catalogue selection: Sign-Less; Number of random selections: 50; P-value/E-value/Q-value/False discovery rate (FDR) (Benjamin-Hochberg corrected) < 0.05; Random control %: 5; and Minimum required number of target genes: 1.

### Statistical analyses

The microarray expression data (signal intensity values) were quantile normalized^56^ and log transformed (base 2). Normal distribution of *COL6A3* expression across the patients was confirmed by Shapiro–Wilk test. Correlations were calculated by Pearson in R. Data from cell culture experiments were analyzed by Mann Whitney U or one-way ANOVA as indicated, using PASW Statistics 18 for Windows.

## Supplementary information

Supplementary Information 1.
