# Supplementary material for: COL6A3 expression in adipose tissue cells is associated with levels of the homeobox transcription factor PRRX1
Source: Sci Rep. 2020 Nov 19;10:20164. doi: 10.1038/s41598-020-77406-2 (PMC7678848; doi:10.1038/s41598-020-77406-2)
Supplement: Supplementary file 1 — Supplementary Information 1. [file 41598_2020_77406_MOESM1_ESM.pdf]

## Supplementary Figures and Tables

*COL6A3 expression in adipose tissue cells is associated with levels of the homeobox transcription factor PRRX1*

Simon N. Dankel[1,2*], Elise Grytten[1,2], Jan-Inge Bjune[1,2], Hans Jørgen Nielsen[3], Arne Dietrich[4], Matthias Blüher[5], Jørn V. Sagen[1,2], Gunnar Mellgren[1,2*]

[1] Mohn Nutrition Research Laboratory, Department of Clinical Science, University of Bergen, Bergen, Norway
[2] Hormone Laboratory, Department of Medical Biochemistry and Pharmacology, Haukeland University Hospital, Bergen, Norway
[3] Department of Surgery, Voss Hospital, Bergen Health Trust, Voss, Norway.
[4] Department of Surgery, University of Leipzig, Germany
[5] Department of Medicine, University of Leipzig, Leipzig, Germany
[*] Corresponding authors: simon.dankel@uib.no and gunnar.mellgren@uib.no

## Contents

**Figure S1** Expression of COL6A3-correlating genes in human adipose tissue and isolated adipocytes and SVF.

**Figure S2** Expression profiles of COL6A3 and PRRX1 during adipogenic differentiation in primary hASCs and 3T3-L1 cells.

**Figure S3** Sequence of the human *COL6A3* promoter in the reporter construct used in Figure 4.

**Table S1.** Sequences of primers and Universal Probe Library (UPL) probes used for qPCR.

**Table S2.** Genes co-expressed with *COL6A3* in subcutaneous adipose tissue from lean and obese people.

**Table S3.** Transcription factors implicated as regulators of *COL6A3* and co-expressed genes in human subcutaneous adipose tissue (TFactS analysis).

**Table S4.** *COL6A3* and co-expressed genes (Pearson's r > 0.75) in subcutaneous adipose tissue, and the transcription factors identified to regulate their expression (TFactS analysis).

## Supplementary Figures

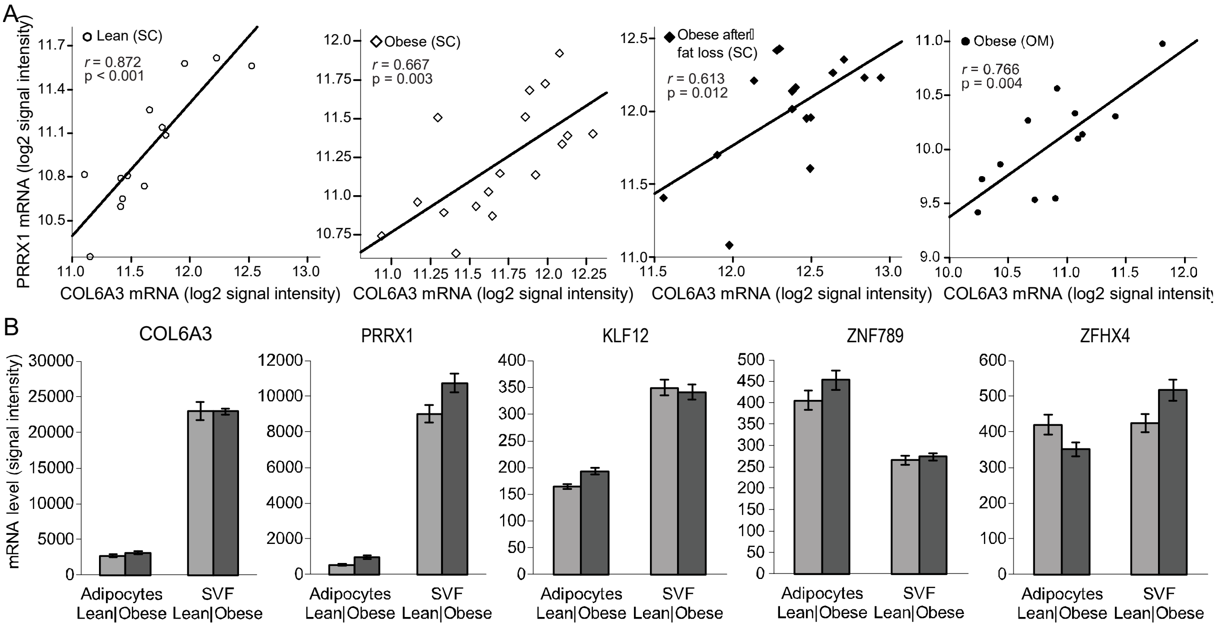


**Figure S1** *Expression of COL6A3-correlating genes in human adipose tissue and isolated adipocytes and SVF*. Gene expression was measured by Illumina microarrays (log2-transformed expression values). **A.** Adipose tissue biopsies were obtained from extremely obese subjects (subcutaneous n=16, omental n=12). Subcutaneous adipose tissue was also obtained from the same subjects after profound fat loss (one year after bariatric surgery, n=16), and from healthy non-obese subjects (n=14). **B.** mRNA expression was measured in paired subcutaneous adipocytes and SVF from morbidly obese people (n=12) and subcutaneous adipocytes from non-obese subjects (n=12). Data are presented as mean ± SD. BMI, body-mass index; SVF, stromal vascular fraction.

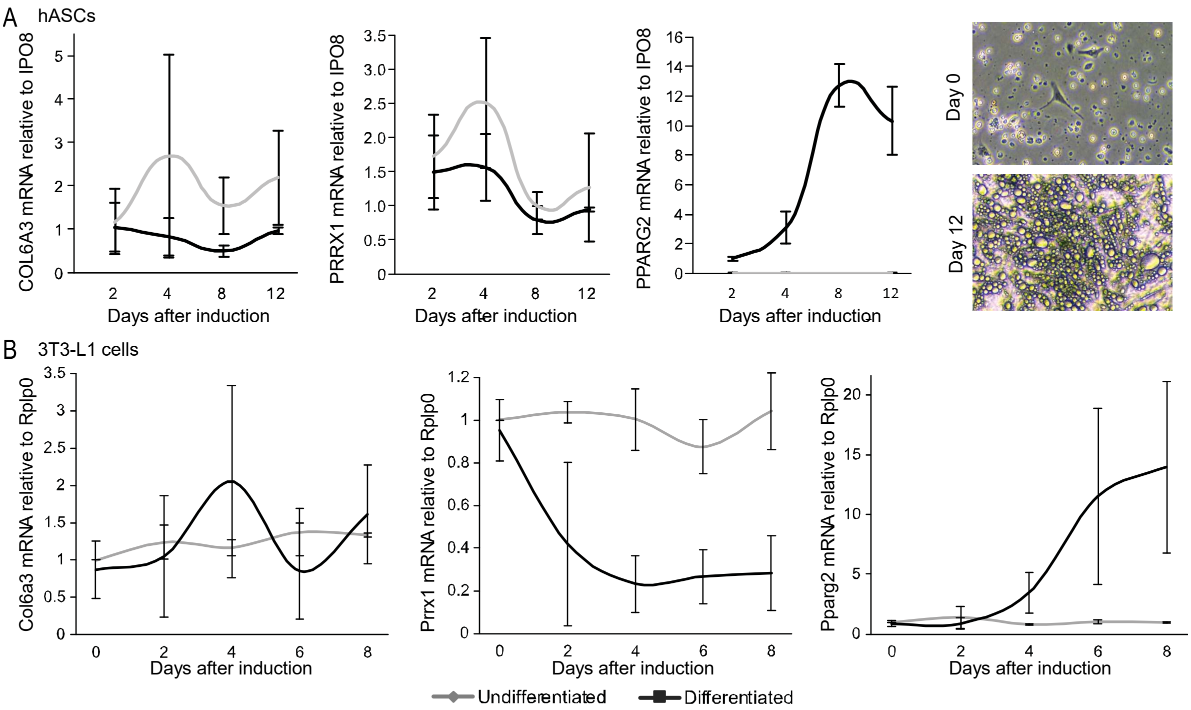


**Figure S2** *Expression profiles of COL6A3 and PRRX1 during adipogenic differentiation in primary hASCs and 3T3-L1 cells.* Cells were induced to differentiate and lysates were collected at the indicated time-points. Target gene expression was measured by qPCR and related to mRNA levels for IPO8 (hASCs, **A**) or Rplp0 (3T3-L1, **B**). Images were taken under the microscope (10x magnification). Data are presented as mean ± SD for four experiments combined (hASCs from four different people, 3T3-L1 for independent experiments). hASC, human adipose stromal cells.

TAAAATGGGACCACAATTTCACCTAATAAAAGAAGACATCTTTCTATTTAAAAGGGCTTAGGGTGTTGATGTTT
GTGATAAAGGAGAGAATGTATATTGAAGTGTTTTGAAATGTGCAAAGCTTTCTAGAAACAGAAGTTCTTACTC
AAGTATTTTCCCGAAGCTTTGGCAAGATAACCATTTTTATTACCCCGTCTGTGCCTAGAATGGGCCTATAAGCG
CCACAATCAGAATCATTAGATATAGAAATTAAGAGAAATGTAGCCTCCTTTTTTTTGCCGGTGAACAGAGCTTT
GGTTAACAGAAAACCAAGGCGATTTTAATTGCTGGTTTTTCTATTTGAAGGGGGAAGTTATTAGTAGAAGTCTC
AATTCAGAAACTTCAAGAAGAAATGGGAGGGTGTGGTGAGGGTAAGCGGGGGACTGCATTTCCTGTTTTCCTT
TCAGATGGTGTTGGAAAACATTGCAGGAAAACCATGGATACCCACGAAGAAATTCCAAAATTTATTCTTTTTGA
CGCCAAGGGCCCAGCCCAAAAGGTGACGAGTAGGAGTGGTCAATTTTTTTTTTTAAGAGTTGGGGCTTGCAGG
AGTCCAGCTAAACGCTTGTAGGGTGAAGACAGAATTCAGAGGGTGACATCAGCCTGAGCGGGGGCCAGAAG
AAACAGAGTGGAGGAGTCTGGTTTCATTTACAGTTTTGGGTCAGTTCTGCAGTGAGGAGGGGGAGAGGAGG
GGTCCGGGAGGGAGGAGGAGGAGGAGGAGGAGCTGGAGGAAGCCCTGACTGGTATCCCTGGCCCCAGTCC
AGTTTGGAGCTCAGTCTTCCACCAAAGGCCGTTCAGTTCTCCTGGGCTCCAGCCTCCTGCAAGGACTGCAAGAG
TTTTCCTCCGCAGCTCTGAGTCTCCACTTTTTTGGTGGAGAAAGGCTGCAAAAAGAAAAAGAGACGCAGTGAG
TGGGAAAAGTATGCATCC

Predicted binding site for PRRX1

**Figure S3** *Sequence of the human COL6A3 promoter in the reporter construct used in Figure 4*. The green highlight shows the predicted PRRX1 binding motif.

## Supplementary Tables

**Table S1.** Sequences of primers and Universal Probe Library (UPL) probes used for qPCR.

| Gene | Forward primer | Reverse primer | UPL probe # |
|---|---|---|---|
| **PRRX1b** | 5’-gtggagcagcccatcgta-3’ | 5’-tgggagggacgaggatct-3’ | 15 |
| **COL6A3** | 5’-cctaaccacatatgttagtggaggt-3’ | 5’-gaatgtctcgcttgctctctg-3’ | 69 |
| **IPO8** | 5’-cggattatagtctctgaccatgtg-3’ | 5’-tgtgtcaccatgttcttcagg-3’ | 48 |
| **HPRT** | 5’-tgaaaaggaccccacgaag-3’ | 5’-aagcagatggccacagaactag-3’ | (SYBR Green) |
| **TBP** | (Roche UPL Human TBP Gene Assay) | | |
| **Prrx1b** | 5’-gagacgtgactgctgtggag-3’ | 5’-aagtagccatggcgctgta-3’ | 32 |
| **Col6a3** | 5’-tcattcccgctgagttcc-3’ | 5’-tgatatcccgtttgtttacgtg-3’ | 32 |
| **Rplp0** | 5’-actggtctaggacccgagaag-3’ | 5’-tcccaccttgtctccagtct-3’ | 9 |

**Table S2.** Genes co-expressed with *COL6A3* in subcutaneous adipose tissue from lean and obese people.

| Gene | Probe ID | Pearson's r | Definition |
|---|---|---|---|
| COL6A3 | ILMN_1706643 | 1 | collagen, type VI, alpha 3, transcript variant 3 |
| COL6A3 | ILMN_2307861 | 0.950195 | collagen, type VI, alpha 3, transcript variant 1 |
| OLFML1 | ILMN_1776151 | 0.881751 | olfactomedin-like 1 |
| PRRX1 | ILMN_1739496 | 0.880918 | paired related homeobox 1, transcript variant pmx-1a |
| COL1A2 | ILMN_2104356 | 0.85236 | collagen, type I, alpha 2 |
| C5orf13 | ILMN_1680738 | 0.851608 | chromosome 5 open reading frame 13 |
| SLC2A10 | ILMN_2120247 | 0.849418 | solute carrier family 2 (facilitated glucose transporter), member 10 |
| PCOLCE | ILMN_1707070 | 0.848099 | procollagen C-endopeptidase enhancer |
| COL3A1 | ILMN_1773079 | 0.847883 | collagen, type III, alpha 1 |
| PDGFRB | ILMN_1815057 | 0.845499 | platelet-derived growth factor receptor, beta polypeptide |
| COL1A1 | ILMN_1701308 | 0.837456 | collagen, type I, alpha 1 |
| KLF12 | ILMN_1714444 | 0.834134 | Kruppel-like factor 12 |
| DCLK1 | ILMN_2165354 | 0.830982 | doublecortin-like kinase 1 |
| PSD3 | ILMN_1717477 | 0.815306 | pleckstrin and Sec7 domain containing 3, transcript variant 1 |
| TRAM1 | ILMN_1737146 | 0.808786 | translocation associated membrane protein 1 |
| GPNMB | ILMN_1801205 | 0.808708 | glycoprotein (transmembrane) nmb, transcript variant 1 |
| C1QTNF6 | ILMN_1729288 | 0.807246 | C1q and tumor necrosis factor related protein 6, transcript variant 1 |
| GLT8D2 | ILMN_1802654 | 0.806704 | glycosyltransferase 8 domain containing 2 |
| PTPRD | ILMN_2315789 | 0.80158 | protein tyrosine phosphatase, receptor type, D, transcript variant 2 |
| MTMR11 | ILMN_1769299 | 0.795634 | myotubularin related protein 11 |
| WDFY1 | ILMN_1676448 | 0.794002 | WD repeat and FYVE domain containing 1 |
| AMMECR1 | ILMN_1779374 | 0.792112 | Alport syndrome, mental retardation, midface hypoplasia and Elliptocytosis chromosomal region, gene 1, transcript variant 1 |
| CXCL12 | ILMN_1791447 | 0.790748 | chemokine (C-X-C motif) ligand 12, transcript variant 1 |
| ZNF789 | ILMN_1789364 | 0.788619 | zinc finger protein 789, transcript variant 1 |
| MMP2 | ILMN_1762106 | 0.785394 | matrix metallopeptidase 2 (72kDa type IV collagenase) |
| MARCKS | ILMN_1807042 | 0.785279 | myristoylated alanine-rich protein kinase C substrate |
| MFAP2 | ILMN_1787981 | 0.78434 | microfibrillar-associated protein 2, transcript variant 1 |
| SH3PXD2A | ILMN_1743103 | 0.78414 | SH3 and PX domains 2A |
| COL5A1 | ILMN_1706505 | 0.779897 | collagen, type V, alpha 1 |
| OLFML3 | ILMN_1727532 | 0.7774 | olfactomedin-like 3 |
| CD47 | ILMN_1771333 | 0.777294 | CD47 molecule, transcript variant 2 |
| FAP | ILMN_2232854 | 0.777257 | fibroblast activation protein, alpha |
| C6orf65 | ILMN_1706969 | 0.776491 | chromosome 6 open reading frame 65 |
| FRMD6 | ILMN_1769282 | 0.773824 | FERM domain containing 6 |
| DPP4 | ILMN_1692535 | 0.773674 | dipeptidyl-peptidase 4 (CD26) |
| KIT | ILMN_2229379 | 0.772317 | v-kit Hardy-Zuckerman 4 feline sarcoma viral oncogene homolog |
| TMEM98 | ILMN_1779182 | 0.769577 | transmembrane protein 98, transcript variant 2 |
| ZFHX4 | ILMN_1657606 | 0.769433 | zinc finger homeobox 4 |
| CA12 | ILMN_1720998 | 0.768223 | carbonic anhydrase XII, transcript variant 1 |
| ST3GAL5 | ILMN_1713496 | 0.767491 | ST3 beta-galactoside alpha-2,3-sialyltransferase 5, transcript variant 2 |
| JARID1B | ILMN_1755727 | 0.767006 | jumonji, AT rich interactive domain 1B |
| CCDC28B | ILMN_2099586 | 0.765988 | coiled-coil domain containing 28B |

| | | | |
|---|---|---|---|
| TMED3 | ILMN_1719316 | 0.764913 | transmembrane emp24 protein transport domain containing 3 |
| DPYSL2 | ILMN_1672503 | 0.764763 | dihydropyrimidinase-like 2 |
| DCN | ILMN_1683194 | 0.7632 | decorin, transcript variant A1 |
| MEX3B | ILMN_1658989 | 0.762618 | mex-3 homolog B (C. elegans) |
| TRO | ILMN_2409642 | 0.761745 | trophinin, transcript variant 6 |
| VASH2 | ILMN_1692698 | 0.75967 | vasohibin 2 |
| FRMD6 | ILMN_2330787 | 0.759559 | FERM domain containing 6, transcript variant 2 |
| MS4A2 | ILMN_1806721 | 0.756646 | membrane-spanning 4-domains, subfamily A, member 2 |
| NT5E | ILMN_1697220 | 0.755628 | 5'-nucleotidase, ecto (CD73) |
| CTSG | ILMN_1680424 | 0.754377 | cathepsin G |
| LPAR1 | ILMN_1701441 | 0.753529 | lysophosphatidic acid receptor 1, transcript variant 2 |
| DPYSL3 | ILMN_1679262 | 0.751755 | dihydropyrimidinase-like 3 |
| PTS | ILMN_2162328 | -0.75365 | 6-pyruvoyltetrahydropterin synthase |
| SBDSP | ILMN_2159384 | -0.75522 | Shwachman-Bodian-Diamond syndrome pseudogene on chromosome 7 |
| ALDH4A1 | ILMN_2406557 | -0.76656 | aldehyde dehydrogenase 4 family, member A1, nuclear gene encoding mitochondrial protein, transcript variant P5CDhS |
| BCL7B | ILMN_2367275 | -0.79011 | B-cell CLL/lymphoma 7B, transcript variant 2 |

Gene expression was measured by Illumina microarrays (see Methods).

**Table S3.** Transcription factors implicated as regulators of *COL6A3* and co-expressed genes in human subcutaneous adipose tissue (TFactS analysis).

| Transcription Factor | P-value | E-value | Q-value | FDR control (B-H) | Intersection | Target genes | Random Control(%) |
|---|---|---|---|---|---|---|---|
| SMAD7 | 0.00E+00 | 0.00E+00 | 0.00E+00 | 1.22E-03 | 4 | 15 | 0 |
| SMAD3 | 1.00E-05 | 4.10E-04 | 5.81E-05 | 2.44E-03 | 4 | 63 | 0 |
| TFAP2A | 9.00E-05 | 3.69E-03 | 3.49E-04 | 3.66E-03 | 4 | 115 | 0 |
| SPI1 | 9.90E-04 | 4.06E-02 | 2.88E-03 | 4.88E-03 | 3 | 93 | 0 |
| NFIC | 4.23E-03 | 1.73E-01 | 8.51E-03 | 6.10E-03 | 2 | 45 | 0 |
| SOX9 | 4.39E-03 | 1.80E-01 | 8.51E-03 | 7.32E-03 | 1 | 2 | 0 |
| ID3 | 6.58E-03 | 2.70E-01 | 9.56E-03 | 8.54E-03 | 1 | 3 | 0 |
| ID2 | 6.58E-03 | 2.70E-01 | 9.56E-03 | 9.76E-03 | 1 | 3 | 0 |
| TCF3 | 1.10E-02 | 4.49E-01 | 1.27E-02 | 1.10E-02 | 1 | 5 | 0 |
| POU1F1 | 1.11E-02 | 4.56E-01 | 1.27E-02 | 1.22E-02 | 2 | 74 | 0 |
| SP1 | 1.20E-02 | 4.91E-01 | 1.27E-02 | 1.34E-02 | 4 | 428 | 4 |
| EP300 | 1.31E-02 | 5.38E-01 | 1.27E-02 | 1.46E-02 | 1 | 6 | 0 |
| MYB | 1.96E-02 | 8.04E-01 | 1.75E-02 | 1.59E-02 | 1 | 9 | 0 |
| MYBL2 | 2.39E-02 | 9.82E-01 | 1.93E-02 | 1.71E-02 | 1 | 11 | 0 |
| HNF1B | 2.61E-02 | 1.07E+00 | 1.93E-02 | 1.83E-02 | 1 | 12 | 0 |
| CTNNB1 | 2.72E-02 | 1.12E+00 | 1.93E-02 | 1.95E-02 | 3 | 306 | 2 |
| POU5F1 | 2.82E-02 | 1.16E+00 | 1.93E-02 | 2.07E-02 | 1 | 13 | 0 |
| TCF7 | 3.68E-02 | 1.51E+00 | 2.38E-02 | 2.20E-02 | 1 | 17 | 0 |
| EBF1 | 3.89E-02 | 1.59E+00 | 2.38E-02 | 2.32E-02 | 1 | 18 | 4 |
| POU2F2 | 4.31E-02 | 1.77E+00 | 2.39E-02 | 2.44E-02 | 1 | 20 | 0 |
| JUNB | 4.31E-02 | 1.77E+00 | 2.39E-02 | 2.56E-02 | 1 | 20 | 0 |
| FLI1 | 5.99E-02 | 2.46E+00 | 3.16E-02 | 2.68E-02 | 1 | 28 | 0 |
| ETV4 | 6.61E-02 | 2.71E+00 | 3.34E-02 | 2.81E-02 | 1 | 31 | 0 |
| NFIA | 7.02E-02 | 2.88E+00 | 3.40E-02 | 2.93E-02 | 1 | 33 | 0 |
| ATF2 | 7.84E-02 | 3.21E+00 | 3.65E-02 | 3.05E-02 | 1 | 37 | 0 |
| MITF | 8.25E-02 | 3.38E+00 | 3.69E-02 | 3.17E-02 | 1 | 39 | 0 |
| YY1 | 9.66E-02 | 3.96E+00 | 4.16E-02 | 3.29E-02 | 1 | 46 | 2 |
| TBP | 1.05E-01 | 4.29E+00 | 4.34E-02 | 3.42E-02 | 1 | 50 | 2 |
| LEF1 | 1.09E-01 | 4.45E+00 | 4.35E-02 | 3.54E-02 | 1 | 52 | 0 |
| ESR1 | 1.13E-01 | 4.61E+00 | 4.36E-02 | 3.66E-02 | 1 | 54 | 0 |
| ATF1 | 1.22E-01 | 5.01E+00 | 4.58E-02 | 3.78E-02 | 1 | 59 | 2 |
| RELA | 1.70E-01 | 6.96E+00 | 6.11E-02 | 3.90E-02 | 1 | 84 | 0 |
| USF2 | 1.73E-01 | 7.11E+00 | 6.11E-02 | 4.02E-02 | 1 | 86 | 0 |
| EGR1 | 1.83E-01 | 7.49E+00 | 6.24E-02 | 4.15E-02 | 1 | 91 | 0 |
| GLI2 | 2.11E-01 | 8.66E+00 | 6.88E-02 | 4.27E-02 | 1 | 107 | 0 |
| USF1 | 2.13E-01 | 8.74E+00 | 6.88E-02 | 4.39E-02 | 1 | 108 | 0 |
| JUN | 2.53E-01 | 1.04E+01 | 7.78E-02 | 4.51E-02 | 1 | 131 | 0 |
| SP3 | 2.54E-01 | 1.04E+01 | 7.78E-02 | 4.63E-02 | 1 | 132 | 0 |
| NFKB1 | 2.69E-01 | 1.10E+01 | 8.02E-02 | 4.76E-02 | 1 | 141 | 0 |
| TP53 | 2.81E-01 | 1.15E+01 | 8.15E-02 | 4.88E-02 | 1 | 148 | 0 |
| FOXO1 | 3.01E-01 | 1.24E+01 | 8.54E-02 | 5.00E-02 | 1 | 161 | 0 |

FDR, false discovery rate.

**Table S4.** *COL6A3* and co-expressed genes (Pearson's r > 0.75) in subcutaneous adipose tissue, and the transcription factors identified to regulate their expression (TFactS analysis).

| | |
|---|---|
| NT5E | ATF1 |
| NT5E | ATF2 |
| CXCL12 | CTNNB1 |
| NT5E | CTNNB1 |
| MMP2 | CTNNB1 |
| CXCL12 | EBF1 |
| COL1A2 | EGR1 |
| COL1A2 | EP300 |
| CXCL12 | ESR1 |
| MMP2 | ETV4 |
| MMP2 | FLI1 |
| DCN | FOXO1 |
| MMP2 | GLI2 |
| DPP4 | HNF1B |
| COL1A2 | ID2 |
| COL1A2 | ID3 |
| COL5A1 | JUN |
| DCN | JUNB |
| NT5E | LEF1 |
| KIT | MITF |
| CTSG | MYB |
| COL1A1 | MYBL2 |
| COL1A2 | NFIA |
| COL1A1 | NFIC |
| COL1A2 | NFIC |
| COL1A1 | NFKB1 |
| PRRX1 | POU1F1 |
| MS4A2 | POU1F1 |
| MS4A2 | POU2F2 |
| KIT | POU5F1 |
| COL1A1 | RELA |
| COL1A2 | SMAD3 |
| COL1A1 | SMAD3 |
| COL3A1 | SMAD3 |
| **COL6A3** | **SMAD3** |

| | |
|---|---|
| COL1A2 | SMAD7 |
| COL3A1 | SMAD7 |
| **COL6A3** | **SMAD7** |
| COL1A1 | SMAD7 |
| COL1A2 | SOX9 |
| MMP2 | SP1 |
| COL1A1 | SP1 |
| COL1A2 | SP1 |
| KIT | SP1 |
| COL1A2 | SP3 |
| PDGFRB | SPI1 |
| MMP2 | SPI1 |
| CTSG | SPI1 |
| COL1A2 | TBP |
| COL1A2 | TCF3 |
| NT5E | TCF7 |
| NT5E | TFAP2A |
| COL1A1 | TFAP2A |
| MMP2 | TFAP2A |
| KIT | TFAP2A |
| MMP2 | TP53 |
| COL1A1 | USF1 |
| COL1A1 | USF2 |
| COL1A1 | YY1 |
